# Detyrosinated α-Tubulin, Vimentin and PD-L1 in Circulating Tumor Cells (CTCs) Isolated from Non-Small Cell Lung Cancer (NSCLC) Patients

**DOI:** 10.3390/jpm12020154

**Published:** 2022-01-25

**Authors:** Spyridoula D. Katsarou, Ippokratis Messaritakis, Anastasia Voumvouraki, Stavros Kakavogiannis, Athanasios Κotsakis, Saad Alkahtani, Christos Stournaras, Stuart S. Martin, Vassilis Georgoulias, Galatea Kallergi

**Affiliations:** 1 Division of Genetics, Cell and Developmental Biology, Department of Biology, University of Patras, 26504 Patras, Greece; med8p1150010@med.uoc.gr (S.D.K.); sstavross1998@gmail.com (S.K.); 2 Department of Zoology, Science College, King Saud University, Riyadh 11451, Saudi Arabia; salkahtani@ksu.edu.sa; 3 Department of Biochemistry, Medical School, University of Crete, 71003 Heraklion, Greece; anastasia180495@gmail.com (A.V.); cstourn@med.uoc.gr (C.S.); 4 Laboratory of Translational Oncology, Medical School, University of Crete, 71003 Heraklion, Greece; imessar@edu.med.uoc.gr; 5 Department of Medical Oncology, University General Hospital of Larisa, 41334 Larisa, Greece; thankotsakis@hotmail.com; 6 Hellenic Oncology Research Group (HORG), 11526 Athens, Greece; georgulv@otenet.gr; 7 Department of Physiology, School of Medicine, University of Maryland, Marlene and Stewart Greenebaum Comprehensive Cancer Center, Baltimore, MD 21201, USA; SSMartin@som.umaryland.edu

**Keywords:** CTCs, lung cancer, alpha-tubulin, detyrosinated α-tubulin, vimentin, PD-L1, metastasis

## Abstract

Upregulation of Vimentin (VIM), alpha-Tubulin (TUB) and Detyrosinated tubulin (GLU) in circulating tumor cells (CTCs) derived from breast cancer patients is related to poor prognosis. In the current study we evaluated for the first time, these cytoskeletal proteins in sixty Non-Small Cell Lung Cancer (NSCLC) patients’ CTCs (33 treatment-naïve and 27 pre-treated). Samples were isolated using the ISET platform and stained with a pancytokeratin (CK)/CD45/TUB, CK/GLU/VIM and CK/programmed death ligand 1 (PD-L1) combination of antibodies. Subsequently, slides were analyzed using confocal laser scanning microscopy. CTCs were detected in 86.7% of the patients. CTCs with TUB expression were identified in 65.4% (34/52) of the CK (+)-patients. GLU, VIM and PD-L1 were also evaluated. The frequency of the observed phenotypes was as follow: (CK+/GLU−/VIM−): 35.2%, (CK+/GLU+/VIM+): 63.0%, (CK+/GLU+/VIM−): 16.7%, (CK+/GLU−/VIM+): 72.2%, (CK+/PD-L1−): 75% and (CK+/PD-L1+): 55%. The OS was significantly decreased in patients with high GLU (3.8 vs. 7.9 months; *p* = 0.018) and/or high VIM (3.2 vs. 7.1 months; *p* = 0.029) expression in their CTCs. PD-L1 was also related to OS (3.4 vs. 7.21 months; *p* = 0.035). Moreover, TUB-high and TUB-low expression in CTCs inversely influenced patients’ OS as independent prognostic factors (*p* = 0.041 and *p* = 0.009). The current study revealed that TUB, GLU, VIM and PD-L1 were overexpressed in CTCs from NSCLC patients. Furthermore, the presence of GLU, VIM-positive and PD-L1 in CTCs is potentially related to patients’ outcomes.

## 1. Introduction

Lung cancer is the second most commonly diagnosed cancer, both in men and women and still remains the leading cause of cancer-related deaths worldwide [1]. Non-Small Cell Lung Cancer (NSCLC) accounts for 85% of all lung cancer subtypes. It is associated with high mortality because usually, the initial diagnosis occurs when the disease is already locally advanced or with distant metastasis [2,3].

It is well known that the presence of circulating tumor cells (CTCs) in the bloodstream is a poor prognostic factor for a number of cancer types, including NSCLC [4,5,6]. CTCs (as part of liquid biopsy) potentially play a key role not only in prognostic assessment but also in early detection of minimal residual disease (MRD) and prediction of the response to treatment strategies [7,8]. It has been proved that CTCs can help to identify the proper therapeutic approach and they can also provide an interesting target for minimizing the metastatic process [8,9,10,11,12]. Knowing the heterogeneity of primary tumors even in patients with the same histologic subtype, CTCs could provide useful information regarding the metastatic potential of different subclones [13].

CTCs that originate from NSCLC are characterized by epithelial-to-mesenchymal transition (EMT) properties, therefore, their detection and characterization are extremely difficult [5,6]. EMT process is responsible for tumor metastasis and invasion of the tumor cells in the bloodstream. It has been shown that cancer cells lose their epithelial phenotype and become more aggressive and capable to create distant metastasis [14,15,16]. The EMT phenotype of CTCs in NSCLC patients is also related to other molecular characteristics, such as epidermal growth factor receptor (EGFR) mutation status, which could lead to a new strategy for targeted therapy [6,17]. Furthermore, it is known that EMT is associated with several immune checkpoint molecules, PD-L1 included. Thus, a number of studies have reported the correlation between EMT status and PD-L1 expression in different types of cancers, including NSCLC [18]. Co-expression of immune checkpoint molecules and vimentin in CTCs could give useful prognostic information for NSCLC patients [19]. We among others have also shown that CTCs derived from NSCLC patients express PD-L1 at baseline level [20]. However, this expression in different stages of the disease evolution and its clinical relevance remains an open issue. Our team has also recently reported that upregulation of α-tubulin (TUB), vimentin (VIM) and detyrosinated α-tubulin (GLU) in CTCs derived from breast cancer patients could be deployed as useful biomarkers to identify cancer cells with an aggressive phenotype. Particularly TUB, GLU and VIM were overexpressed in metastatic compared to early breast cancer patients’ CTCs and the detection of CK+/GLU+/VIM+ tumor cells in peripheral blood was correlated to shorter Progression Free Survival (PFS) [21]. It is proved that all these molecules support microtentacle formation and participate in the metastatic cascade as a mechanism of dissemination [22,23]. According to our previous results, microtentacle protrusions also participate in inter–CTCs communication and to the potential crosstalk between CTCs and blood cells [21].

However, the expression of these proteins has not been studied yet in CTCs derived from NSCLC patients. Therefore, to assess the universal role of these molecules in different types of cancers, we investigated the expression pattern of TUB, GLU and VIM in CTCs detected in metastatic NSCLC patients and analyzed their potential prognostic value.

## 2. Materials and Methods

### 2.1. Cancer Cell Lines

In order to define the expression pattern of TUB, GLU and VIM in patients’ samples we used the breast cancer cell line MDA-MB 231 (metastatic breast cancer, as the positive control according to our previous study [12]), as well as the following lung cancer cell lines: H1299 (lymph node metastasis of a lung adenocarcinoma), SKMES (squamous cell carcinoma), H460 cell line (large cell lung cancer) and HCC827 (adenocarcinoma lung cancer). All cell lines were obtained from the ATCC (American Type Culture Collection, Manassas, VA, USA). The MDA-MB 231 cells were cultured in RPMI Medium 1640 (GIBCO-BRL Co, MD, USA) supplemented with 10% fetal bovine serum (FBS; GIBCO-BRL Co, MD, USA) and 50 mg/mL penicillin/streptomycin. The H1299 cell line was cultured in 1:1 Dulbecco’s Modified Eagle Medium (GIBCO-BRL Co, MD, USA) supplemented with 10% FBS (GIBCO-BRL Co, MD, USA) and 50 mg/mL penicillin/streptomycin. The SKMES cell line was cultured in MEM-alpha (GIBCO-BRL Co, MD, USA) with 10% FBS and 50 mg/mL penicillin/streptomycin. The HCC827 cell line was cultured in RPMI (GIBCO-BRL Co, MD, USA) plus 10% FBS and penicillin/streptomycin. Finally, the H460 cell line was cultured in RPMI, 5% sodium pyruvate and 10% FBS and penicillin/streptomycin. Every cell line was used in control experiments between the 2nd and 10th passages to reassure the stable cell line profile. Subcultivation for all cell lines was performed with 0.25% trypsin and 5 mM ethylenediaminetetraacetic acid (EDTA). Cells were maintained in a humidified atmosphere of 5% CO_2_/95% air and all experiments were performed during the logarithmic growth phase.

### 2.2. Patients’ Blood Samples

Peripheral blood (20 mL in EDTA) was obtained from 60 patients with stage IV NSCLC before the initiation of any line of treatment. Thirty-three patients were newly diagnosed (treatment-naïve) and 27 had received at least one previous line of treatment. All the patients’ characteristics are shown in Supplemental Table S1. Blood samples were collected at the middle of vein puncture after the first 5 mL of blood were discarded to avoid contamination of the blood sample with epithelial cells from the skin during sample collection. The study complied with the Ethical Principles for Medical Research Involving Human Subjects according to the World Medical Association Declaration of Helsinki and was approved by the local ethics and scientific committees of the University Hospital of Heraklion, Greece (No.20068—30 January 2015). All patients provided written informed consent to participate in the study.

### 2.3. ISET Isolation System of CTCs

CTCs were isolated using the ISET (Isolation by Size of Tumor cells) platform (Rarecells Diagnostics, Paris, France) according to the manufacturer’s instructions. Briefly, 10 mL of peripheral blood were diluted in 1:10 ISET buffer (Rarecells Diagnostics, Paris, France) for 10 min at room temperature (RT), and 100 mL of the diluted sample was filtered using the depression tab adjusted at 10 kPa. The membrane was dried for 2 h at RT and stored at −20 ℃. Each membrane spot was used for the identification of CTCs after immunostaining and confocal laser scanning microscopy analysis.

### 2.4. Immunofluorescence Staining and Confocal Laser Scanning Microscopy

Patients’ samples were analyzed for the expression of TUB, GLU and VIM using triple immunofluorescence (IF) staining with the following combinations of antibodies (Abs): CK/TUB/CD45 and CK/GLU/VIM. Thereafter, all samples were evaluated using a confocal laser scanning microscope module (Leica Lasertechnik, Heidelberg, Germany). Intensity per pixel was analyzed using Image J software.

For triple staining CK/TUB/CD45, spots were incubated with phosphate buffered saline solution (PBS) for 5 min and then cells were permeabilized with 0.2% Triton for 10 min. After 1 h blocking with PBS/10% FBS, spots were incubated with TUB anti-mouse Ab (Cell Signaling Technology, Danvers, MA, USA) for 1 h, followed by Alexa Fluor 555 anti-mouse secondary Ab (Invitrogen Molecular Probes, Eugene, OR, USA) for 45 min. Subsequently, Zenon technology (Fluorescein Isothiocyanate (FITC)-conjugated IGg1 antibody) (Invitrogen Molecular Probes, Eugene, OR, USA) was used for CK detection. For Cytokeratin staining A45-B/B3 anti-mouse Ab recognizing the CKs 8,18,19 (Micromet Munich, Germany) and an anti-mouse Ab against CK7 (Abcam, Cambridge, UK) was used. Zenon reagent was incubated with A45-B/B3 and CK7 cocktail antibodies for 5 min, blocking buffer was added for 5 min and the conjugated Abs were then ready for use. Zenon antibodies were prepared within 30 min before use and were added to the samples for 1 h. Cells were also stained with an anti-CD45 antibody (common leukocyte antigen) conjugated with Alexa 647 (Novus Biologicals, Abingdon, UK), in order to exclude the possible ectopic expression of cytokeratins by hematopoietic cells. Finally, cells were stained with 4′,6-diamidino-2-phenylindole (DAPI; Invitrogen, Carlsbad, CA, USA) conjugated with antifade. Considering that CTCs derived from NSCLC have very low expression of CK other cytomorphological criteria by Hofman and colleagues, such as the existence of an irregular nucleus, high nuclear to cytoplasmic ratio, multiple and large nucleoli, the large size of the cell (>24 μm), etc., were also used in order to characterize tumor cells in the samples [24,25].

Triple IF for CK/GLU/VIM was also performed. The aforementioned methodology for permeabilization and blocking was followed and then the spots were stained with GLU anti-rabbit Ab (Abcam, Cambridge, MA, USA) overnight at 4 °C. Subsequently, cells were incubated with Alexa Fluor 633 anti-rabbit secondary antibody (Life Technologies, Carlsbad, CA, USA) for 45 min. Consequently, cells were incubated for 1 h with VIM anti-mouse Ab (Santa Cruz Biotechnology, Dallas, Texas, USA) and stained with Alexa Fluor 555 anti-mouse secondary Ab (Invitrogen, Carlsbad, CA, USA). Finally, Zenon technology (Invitrogen, Carlsbad, CA, USA) was used for CK detection with the A45-B/B3/CK7 cocktail antibodies, following the procedure mentioned before and cells were stained with DAPI (Invitrogen, Carlsbad, CA, USA) conjugated with antifade.

For PD-L1/CK staining cells were permeabilized with 2% Triton for 10 min. After 1 h blocking with PBS/10% FBS, spots were incubated with anti-PD-L1(clone B7-H1/PD-L1/CD274; Novus Biologicals, Abingdon, UK) antibody. For the detection of PD-L1, the samples were further incubated with Alexa 555 anti-rabbit antibody (Invitrogen, Carlsbad, CA, USA). Consequently, A45-B/B3/CK7 pancytokeratin antibodies were added and cells were incubated with FITC secondary antibody (Invitrogen, Carlsbad, CA, USA). Finally, DAPI was added for the visualization of the nucleus.

A patient is considered positive for a phenotype if he has at least one CTC that belonged to this phenotype.

Positive controls were included in each experiment using the above cell lines, while negative controls were prepared by omitting the corresponding primary antibodies incubating the cells with the matching immunoglobulin (Ig)G isotype bound to the corresponding fluorochrome. Each patient with at least one CTC belonging to a distinct phenotype was considered as positive for this phenotype.

### 2.5. Statistical Analysis

Overall survival (OS) was defined as the time from enrollment into the study until death from any cause. Progression-free survival (PFS) was defined as the time from enrollment until disease relapse or death, whichever occurred first. Kaplan–Meier curves and Cox regression analysis for PFS and OS were compared using the log-rank test or Breslow test to provide a univariate assessment of the prognostic value of selected clinical risk factors. Cox proportional hazards regression model was used to identify those with independent prognostic values. All statistical tests were performed at the 5% level of significance. IBM SPSS Statistics version 22 software (IBM, Armonk, NY, USA) was used for the analysis.

## 3. Results

### 3.1. Evaluation of TUB, GLU and VIM in Lung Cancer Cell Lines

**Figure 1:**
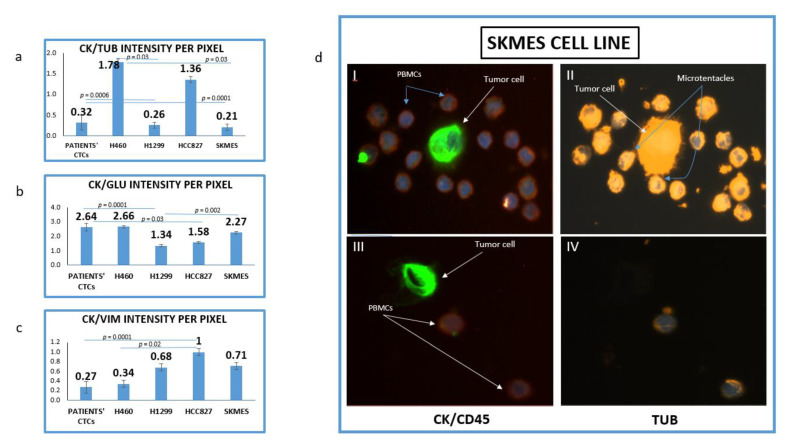
Expression of Cytokeratin (CK), alpha-Tubulin (TUB), Detyrosinated Tubulin (GLU) and vimentin (VIM) in lung cancer cell lines. (**a**) Quantification of (CK/TUB ratio) intensity per pixel in Lung cancer cell lines using Image J software; (**b**) Quantification of (CK/GLU ratio) intensity per pixel in Lung cancer cell lines, using Image J software; (**c**) Quantification of (CK/VIM ratio) intensity per pixel in Lung cancer cell lines, using Image J software; (**d**) Representative image of SKMES lung cancer cells spiked in normal blood donors’ PBMCs, obtained from Fluorescence microscope of patients’ CTCs (×40). (I, II) SKMES cells positive for Cytokeratin (green) negative for CD45 (red), positive for Tubulin (orange) and DAPI (blue) and (III, IV) SKMEs cell positive for CK (green), negative for CD45 (red) and negative for TUB.

The mean intensity per pixel for all the examined molecules (TUB, GLU and VIM) was evaluated in NSCLC cell lines and patients’ CTCs. It was interesting that there was a great variation regarding TUB and GLU expression not only between cell lines but also among cells in the same cell line. Figure 1d is showing two different SKMES lung cancer cells. The first cell (Figure 1d, I–II) expressed a high level of TUB, however, the second one (Figure 1d, III–IV) was negative for TUB. Interestingly in the TUB positive cells, microtentacles of tubulin participate in the communication between cancer cells and normal peripheral blood mononuclear cells (PBMCs) (blue arrows).

The highest expression of TUB was observed in the metastatic H1299 cell line (CK/TUB ratio: 0.26 ± 0.072, Figure 1a). The mean expression of TUB in CTCs was also high (CK/TUB ratio: 0.32 ± 0.18) comparable to NSCLC cell lines. In accordance with these results, the mean expression of GLU in H1299 was the highest among cell lines (CK/GLU ratio: 1.34 ± 0.08, Figure 1b), while the mean intensity in patients CTCs was (CK/GLU ratio: 2.75 ± 0.27).

The highest expression of Vimentin was observed in H460 (CK/VIM ratio: 0.34 ± 0.08, Figure 1c). Interestingly patients’ CTCs revealed higher expression of Vimentin than any cell line (CK/VIM ratio: 0.27 ± 0.12).

The lowest positivity was observed for both TUB (1.78 ± 0.07) and GLU (2.75 ± 0.08) in the H460 cell line and this was used as a cut-off value for positivity of these proteins in CTCs. The lowest expression of vimentin was observed in HCC827 (1 ± 0.06).

### 3.2. Detection of TUB in CTCs Derived from NSCLC Patients

Among the 60 examined patients’ samples, CTCs (CK+/CD45− cells) were observed in 52 (86.7%). The mean number of CTCs per patient was 6.32.

**Figure 2:**
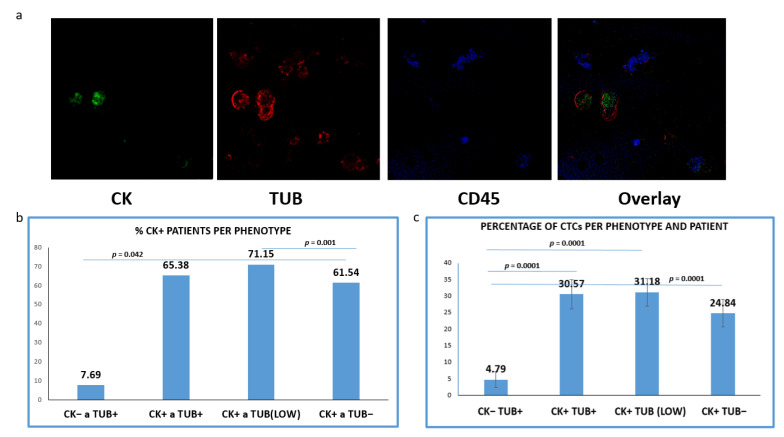
Expression of Cytokeratin (CK) and alpha-Tubulin (TUB) in patients’ CTCs. (**a**) Representative confocal laser scanning micrographs of patients’ CTCs (×40) stained for CK (green), Tubulin (red), CD45 (blue) and DAPI (not shown because our confocal has no laser for DAPI); (**b**) Percentages of patients belonging to distinct phenotypes. Each patient was considered as positive for a distinct phenotype, if he/she is harboring at least one CTC with this phenotype; (**c**): Percentage of CTCs per patient and per phenotype in the whole examined group of subjects.

CTCs positive for TUB were detected in 65.38% of the CK+ patients (34 out of 52), whereas tumor cells with low levels of TUB expression were identified in 71.15% (37/52) of the patients. Meanwhile, 61.54% (32/52) of the samples harbored CTCs characterized by the complete absence of TUB expression (Figure 2b). Interestingly, 7.69% (4/52) of the patients (only with advanced disease) harbored CTCs with (CK−/TUB+CD45−) phenotype.

Subsequently, we evaluated the average percentages of CTCs per phenotype and per patient according to our previous studies [20]. This percentage was calculated as the average of the percentages of this phenotype in all CK-positive patients. Among the total isolated CTCs, 30.57% were (CK+/TUB+CD45−), while the rest of the cells appeared with either low (35.97%) or negative (28.66%) tubulin expression (Figure 2c); 4.79% of the total isolated CTCs (only in advanced disease) belonged to (CK−/TUB+CD45−) phenotype and the frequency of these tumor cells was statistically different compared to all the other phenotypes (Figure 2c).

Representative photos (Figure 2a) of intracellular distribution of CK, TUB and CD45 in a patient’s sample show a heterogeneous expression of CK. It is obvious that the expression of CK was rather low in tumor cells, while CTCs with complete absence of CK (CK−/TUB+CD45−) were also present.

Analysis of patients’ samples regarding their treatment stage (treatment naïve vs. pre-treated patients) showed that 31 out of 33 treatment naive patients (93.9%) were positive for CTCs, while 21 out of 27 (77.8%) pre-treated patients had CK-positive CTCs.

**Figure 3:**
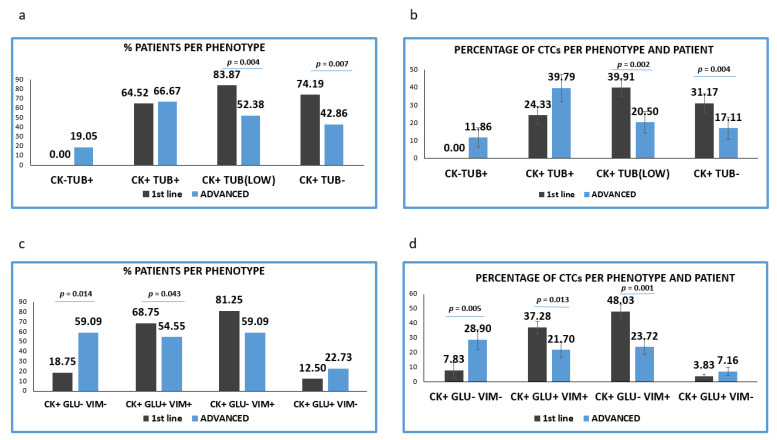
Expression of Cytokeratin (CK), Detyrosinated α-tubulin (GLU) and Vimentin (VIM) in treatment naïve versus pre-treated patients’ samples. (**a**) Percentage of patients belonging to discrete CTCs’ phenotype, regarding the CK/TUB/CD45 staining in treatment naïve (1st line) versus pre-treated (advanced) patients. Each patient was considered as positive for a distinct phenotype, if he/she’s harboring at least one CTC with this phenotype; (**b**) Percentage of CTCs per patient and per phenotype in treatment naïve (1st line) versus pre-treated (advanced) patients, regarding the CK/TUB/CD45 staining; (**c**) Percentage of patients belonging to discrete CTCs’ phenotype, regarding the CK/GLU/VIM staining, in treatment naïve (1st line) versus pre-treated (advanced) patients. Each patient was considered as positive for a distinct phenotype if he/she’s harboring at least one CTC with this phenotype; (**d**) Percentage of CTCs per patient and per phenotype in treatment naïve (1st line) versus pre-treated (advanced) patients, regarding the CK/GLU/VIM staining.

CTCs with high TUB expression were observed in almost equal proportions of patients in the two treatment settings [(64.52% (20/31) vs. 66.67% (14/21) respectively]. CTCs negative or with low expression of TUB were more frequent in previously untreated patients. Particularly low TUB expression was detected in 83.87% (26/31) in patients before the initiation of 1st line treatment versus 52.38% ((11/21), *p* = 0,004) in pre-treated patients. Furthermore, TUB negative CTCs were detected in 74.19% (23/31) vs. 42.86% (9/21), (*p* = 0.007) respectively (Figure 3a).

The proportion of TUB low or negative CTCs isolated from the two cohorts of patients was statistically higher in treatment-naïve, compared to pre-treated patients (*p* = 0.002 and *p* = 0.004, respectively). Conversely, the proportion of TUB high CTCs was found to be increased in pretreated compared to treatment-naive patients (35.56% vs. 23.37%) (Figure 3b).

### 3.3. Detection of GLU and Vimentin in CTCs Derived from NSCLC Patients

**Figure 4:**
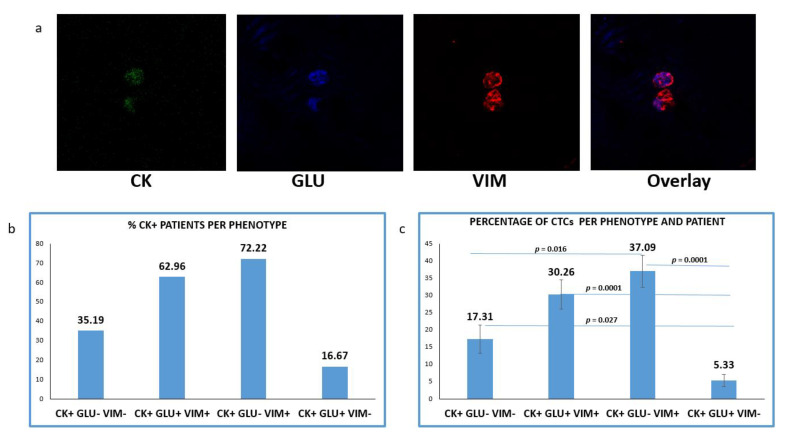
Expression of Cytokeratin (CK), Detyrosinated α-tubulin (GLU) and Vimentin (VIM) in patients’ CTCs. (**a**) Representative confocal laser scanning micrographs of patients’ CTCs (×40) stained for CK (green), VIM (red), GLU (blue) and DAPI (not shown because our confocal has no laser for DAPI); (**b**) Percentages of patients belonging to distinct phenotypes. Each patient was considered as positive for a distinct phenotype, if he/she’s harboring at least one CTC with this phenotype; (**c**) Percentage of CTCs per patient and per phenotype in the whole examined group of subjects.

One additional spot from the same cohort of patients was simultaneously evaluated for CK/GLU/VIM (Figure 4a). CTCs in this staining was detected in 54 out of 60 (90%) NSCLC patients. The mean number of CTCs per patient was 4.18.

CTCs positive for GLU with a concurrent expression of VIM (CK+/GLU+/VIM+) were observed in 62.96% (34 of 54) of CK-positive patients. Tumor cells with (CK+/GLU+/VIM−) phenotype were detected in 16.67% (9 of 54) of the patients’ samples. CTCs lacking expression of both molecules (CK+/GLU−/VIM−) were detected, in 35.19% (19/54) of the subjects (Figure 4b).

Among the total isolated CTCs, the highest proportion (41.21%) belonged to (CK+/GLU−/VIM+) phenotype. The next very frequent phenotype was the (CK+/GLU+/VIM+), with the proportion of these cells reaching 33.63%. Smaller percentages of CTCs characterized by lack of VIM (5.92%) or lack of both VIM and GLU followed (19.24%) (Figure 4c).

Analysis of the different phenotypes in treatment naïve versus pretreated patients, revealed that the percentage of patients’ samples harboring the (CK+/GLU−/VIM+) and (CK+/GLU+/VIM+) phenotypes were higher in 1st line patients (81.25% vs. 59.09%, respectively; *p* = 0.192) and (68.75% vs. 54.55%, respectively; *p* = 0.043) (Figure 3c).

The percentage of patients harboring (CK+/GLU−/VIM−) tumor cells were found to be statistically higher in pre-treated compared to treatment-naïve patients (18.75% vs. 59.09% *p* = 0.014) (Figure 3c).

Assessment of the proportion of the total examined CTCs per patients and per phenotype (Figure 4d) revealed that the CTCs belonged to (CK+/GLU+/VIM+) and (CK+/GLU−/VIM+) phenotypes were enhanced in treatment-naïve compared to pre-treated patients ((38.44% vs. 27.21, respectively; *p* = 0.013) and (49.53% vs. 30.45%, respectively; *p* = 0.001)). Conversely, the phenotype (CK+/GLU−/VIM−) was increased in pre-treated patients ((8.07% vs. 33.93%, respectively; (*p* = 0.005)). Finally, the phenotype (CK+/GLU+/VIM−) was the least common in both settings (3.95% vs. 8.41%, respectively).

**Figure 5:**
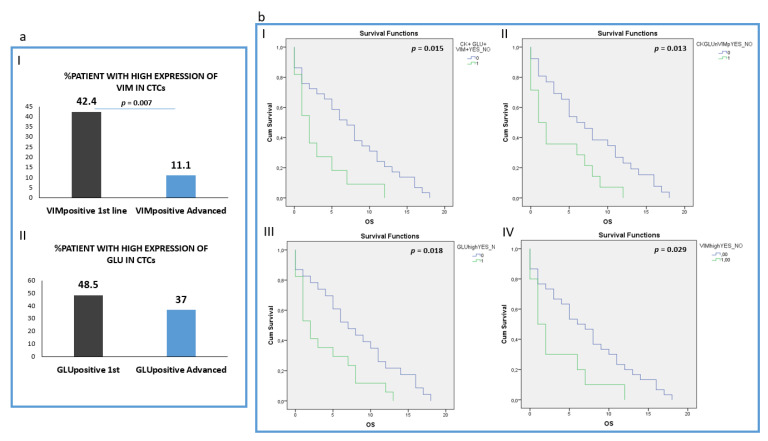
Clinical outcome in NSCLC patients. (**a**) (I) Percentage of treatment naïve (1st line) versus pre-treated (advanced) patients with high VIM expression in their CTCs; (II) Percentage of treatment naïve (1st line) versus pre-treated (advanced) patients with high GLU expression in their CTCs; (**b**) (I) Overall survival (OS) in patients harboring the (CK+GLU+VIM+) phenotype in their CTCs; (II) Overall survival (OS) in patients harboring the (CK+GLU-VIM+) phenotype in their CTCs; (III) Overall survival (OS) in patients harboring High expression of GLU in their CTCs; (IV) Overall survival (OS) in patients harboring High expression of VIM in their CTCs.

Analysis of the intensity of the two molecules (Vimentin and GLU) revealed that patients harboring CTCs with high VIM expression (higher or equal to H460 cell line) were found more frequently in 1st line compared to pre-treated patients (42.4% (14 out of 33) vs. 11.1 (3 out of 27), respectively; *p* = 0.007) (Figure 5a, I). However, the percentage of patients with high GLU expression in their CTCs (higher or equal to H1299) was not statistically different between the two groups: 48.5% (16 out of 32) in treatment-naïve versus 37% (10 out of 27) in pre-treated patients (Figure 5a, II). The absolute number of CTCs per patient for each distinct phenotype is shown in Supplemental Table S2.

### 3.4. Detection of PD-L1 in CTCs Isolated from NSCLC Patients

**Figure 6:**
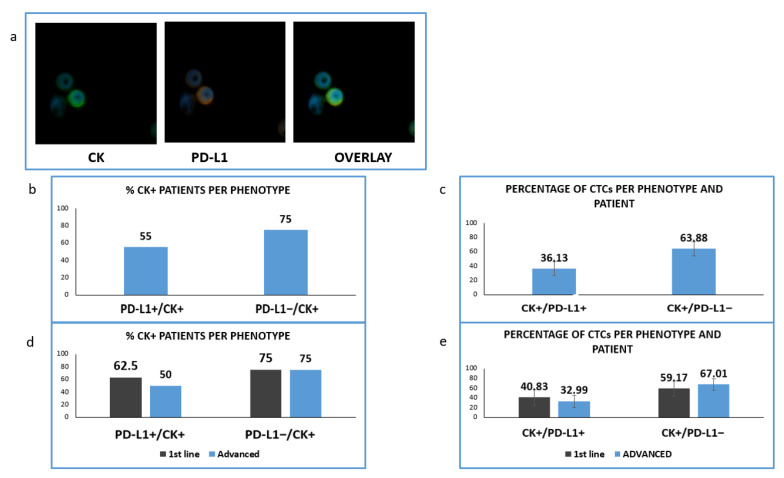
Expression of Cytokeratin (CK) and PD-L1 in patients’ samples. (**a**) CK (green) and PD-L1 (red) staining in patients’ samples; (**b**) Percentage of patients with PD-L1-positive and PD-L1 negative CTCs; (**c**) Percentage of CTCs-expressing PD-L1 among the total isolated tumor cells; (**d**) Percentage of patients expressing PD-L1 among the treatment naïve (1st line) versus pre-treated (advanced) patients; (**e**) Percentage of CTCs-expressing PD-L1 in treatment naïve (1st line) versus pre-treated (advanced) patients.

Consequently, we have evaluated, in the same group of patients with available samples, an immune checkpoint molecule; PD-L1 protein. Particularly, samples from 42 (16 untreated and 26 pretreated) patients were double stained for CK/PD-L1 (Figure 6).

We found that in this cohort of patients (CK-positive cells could be identified in 20 of them. PD-L1 was expressed in CTCs isolated from 11 out of 20 CK-positive NSCLC patients (55%) and PD-L1-negative CTCs were detected in 15 out of 20 (75%) (Figure 6b). The corresponding percentages of CTCs were 36.13% and 63.88% (Figure 6c).

Analyzing the distribution of distinct phenotypes in the two examined groups of patients, PD-L1-positive CTCs were found in five out of eight (62.5%) and in six out of 12 (50%) of CK-positive 1st line and pretreated patients respectively (Figure 6d). Finally, PD-L1-negative CTCs were detected in six out of eight (75%) and nine out of 12 (75%) correspondingly (Figure 6d). The percentage of PD-L1 positive CTCs among the total number of isolated tumor cells was 40.83% vs. 32.99% for 1st line and advanced disease (Figure 6e).

### 3.5. Clinical Outcome According to TUB, GLU and VIM Expression

Clinical data for 40/60 patients were available for further evaluation. After a median follow up of 5 months (range, 0–18), all the patients died. The median PFS of the patients was 2 months (range 0–16).

Analysis of all the different phenotypes revealed that patients harboring CTCs with the (CK+/GLU+/VIM+) phenotype experienced shorter OS (*p* = 0.015, Log Rank test, HR: 2.33, (3.1 vs. 7.31 months)), (Figure 5b, I).

In addition, patients harboring the (CK+/GLU−/VIM+) phenotype in their CTCs, revealed poorer OS (*p* = 0.013, Log Rank test, HR: 2.27, (3.50 vs. 7.57 months)) (Figure 5b, II).

Furthermore, patients with high expression of GLU in their CTCs regardless of the expression of the rest of the examined molecules also revealed poorer overall survival (*p* = 0.018, Log Rank, HR: 2.13, (3.8 months vs. 7.8 months,)) (Figure 5b, III). Patients also with high Vimentin expression in their CTCs experienced shorter OS (*p* = 0.029, Log Rank, HR: 2.18, (3.2 vs. 7.1 months)) (Figure 5b, IV).

In line with GLU and VIM results, the expression of PD-L1 in CTCs was related to poorer OS (*p* = 0.035, 3.4 vs. 7.2 months) (Supplemental Figure S1e).

The expression of TUB in CTCs was potentially related to shorter OS (*p* = 0.027, Breslow test, HR: 1.58, (4.4 months vs. 7.9 months)), (Supplemental Figure S1a).

Interestingly, analysis of the total number of CTCs irrespective of their phenotypic characterization and disease status, revealed that the presence of CTCs was not significantly correlated to patients’ outcomes, implying that the characterization and not the enumeration of these cells is important for patients’ prognosis (*p* = 0.860, Log Rank, (6.17 vs. 6.15 months)).

Analysis of the treatment naïve patients revealed that high expression of tubulin was related to poorer OS in this stage of the disease (*p* = 0.019, Log Rank, HR: 3.57, (3 vs. 7.5 months)). (Supplemental Figure S1b).

In pre-treated patients, the detection of low expression of TUB in CTCs was related to a better outcome (*p* = 0.042, Log Rank, HR: 0.42, (4.6 vs. 10.44 months)), (Supplemental Figure S1c).

Finally, in this cohort of patients, the presence of CTCs with high expression of Vimentin was related to shorter OS (*p* = 0.019, Log Rank, HR: 5.12, (0.5 vs. 7.52 months)) (Supplemental Figure S1d).

Multivariate analysis revealed that the presence of TUB high-CTCs and TUB low- CTCs in patients’ blood were independent prognostic factors for OS (*p* = 0.041 HR:2.6 and *p* = 0.009 HR:0.285).

## 4. Discussion

The enumeration and characterization of Circulating Tumor Cells is an important prognostic marker for NSCLC patients [5,24]. It is also widely accepted that CTCs could undergo Epithelial to Mesenchymal Transition in many different types of cancers, such as breast, colon, NSCLC, SCLC, etc., [26,27,28,29,30,31,32]. Particularly, the expression of epithelial markers in CTCs derived from NSCLC patients is extremely low, thus their detection based on common epithelial antigen makes them invisible in the bloodstream [5,25]. However, despite these difficulties, the investigation of the distinct molecular characteristics and properties of CTCs is very important for patients [8].

Another important step in understanding the mechanism of the metastatic process is the identification of the role of the crosstalk between immune cells and CTCs [33]. We among others have recently shown that CTCs express immune checkpoint molecules, implying an interaction between tumor and immune cells in the bloodstream. [20,34,35]. However, this crosstalk involves different aspects of communication. In particular, we have recently shown that circulating tumor cells can potentially be in physical contact with immune cells, through membrane filamentous bridges (microtentacles) [21]. These membrane protrusions supported by alpha TUB, GLU and VIM have been shown to participate in the metastatic process. Cancer cells with an aggressive phenotype express an increased number of microtentacles that facilitate the migration and invasion of these cells [21,22,23]. Furthermore, we have shown that overexpression of these molecules in CTCs could be related to prognostic significance in breast cancer patients. However, the expression of these molecules in CTCs derived from NSCLC patients had not been studied so far.

In the current study, we investigated the expression of TUB, GLU and VIM in NSCLC cell lines (as controls) and in CTCs isolated from stage IV NSCLC patients. Using four different lung cancer cell lines (H460, H1299, HCC827 and SKMES) we created an expression pattern of these molecules. Interestingly, we observed an important variation regarding TUB and GLU expression not only between different lung cancer cell lines but also among the cells of a distinct cell line (Figure 1d). Tumor cells that expressed alpha tubulin were often in touch with PBMCs, through membrane protrusions (microtentacles) consisting of alpha tubulin. These microtentacles participate in this crosstalk, implying a critical role in the interaction between tumor and immune cells in the bloodstream [21].

We have also performed IF experiments in all patients’ samples and analyzed the clinical relevance of these data. We among others have recently shown that the ISET system could help to obtain a high recovery rate of CTCs [5,36], therefore, we used this technology to isolate CTCs from NSCLC patients. The limitation of this isolation method is the exclusion of CTCs smaller than 8 μΜ, however, according to our studies and to available bibliography, it offers a better recovery rate compared to other methods, such as CellSearch, Ficoll density gradient, etc., [5,24,25,36]. This attributes to the fact that CTCs with mesenchymal characteristics are not omitted, due to size-based isolation of tumor cells, regardless of their EMT status. In agreement with previous reports, CTCs in the current study could be detected in up to 90% of NSCLC patients. In line with previous studies regarding EMT in NSCLC patients, we noticed (Figure 2a and Figure 4a) that Cytokeratins were downregulated in CTCs [5,26]. Particularly, tumor cells with a complete absence of cytokeratin expression (CK−/TUB+/CD45−) were detected in 19.05% of the pretreated patients (Figure 3a).

The evaluation of TUB expression in CTCs revealed that the phenotype (CK+/TUB+/CD45−) was more frequent in patients with advanced disease (Figure 3b). This observation is in line with results obtained from our previous study in breast cancer patients. In this work, the expression of TUB was increased in the metastatic setting, reinforcing the assumption that TUB is related to the metastatic process and tumor progression [21]. This expression was related to poorer OS in the whole cohort of patients (*p* = 0.027, 4.4 vs. 7.9 months). Moreover, low tubulin expression was observed mainly in treatment naïve compared to pretreated patients (83.87% vs. 52.38%, *p* = 0.004). The same observation was applied for patients harboring TUB-negative CTCs (74.19% vs. 42.86%, *p* = 0.007). Interestingly, the presence of CTCs with low TUB expression in advanced disease was related to better OS (*p* = 0.042, 4.6 vs. 10.44 months), while upregulated TUB in treatment naïve was related to poorer OS (*p* = 0.019, 3 vs. 7.5 months). These findings were expected, since TUB supports the formation of microtentacles, which increase the migratory capacity of the cells, playing a key role in adherence of cancer cells to distant tissue and thus to metastatic growth [22,37,38]. Multivariate analysis revealed also that TUB high *(p* = 0.041 HR:2.6) and TUB low expression in CTCs (*p* = 0.009 HR:0.285) was related to poorer and better outcomes of the disease as independent prognostic factors, respectively.

Investigation of GLU expression in the same cohort of patients revealed that this post-translational modification of alpha TUB was present in all the examined disease stages and high expression of GLU in the whole cohort of patients was related to poorer OS (*p* = 0.018 3.8 months vs. 7.8 months), (Figure 5b, III). This observation is in agreement with previous studies reporting that the presence of GLU in primary tumors could be a poor prognostic factor [39]. It is also in line with the results from our previous study in breast cancer patients, where the presence of CK+/GLU+/VIM+ phenotype was a poor prognostic factor for metastatic patients. This phenotype is also related to poorer OS in NSCLC patients (*p* = 0.015, 3.1 vs. 7.31 months), (Figure 5b, I).

Finally, VIM highly expressing CTCs were statistically more frequently observed in treatment naïve (*p* = 0.007, Figure 6a, I) compared to pretreated patients, implying that these CTCs could be affected by therapeutic treatment in agreement with other studies [28,39]. Alternative it is possible that the expression of vimentin precedes GLU conversion during EMT. This is reinforced by the fact that the highest proportion (81.3%) of patients belonged to (CK+/GLU−/VIM+) phenotype in treatment naïve patients, while the phenotype (CK+/GLU+/VIM−) was less frequent in both groups. Despite this fact, the expression of this EMT marker both in the whole patient group and in pretreated patients was a poor prognostic factor. (OS: *p* = 0.029 (3.2 vs. 7.1 months) and *p* = 0.019 (0.5 vs. 7.52 months) respectively), (Figure 5b, IV, Supplemental Figure S1d). This observation is in line with previous studies showing that the expression of vimentin in CTCs from NSCLC patients is related to more aggressive disease with liver metastasis and poor prognosis [15,16,40]. These results are also in line with our previous study and with other studies in different cancer types, such as colon, prostate, etc., [21,29,41,42].

It has been recently proved that EMT and immune evasion are related to poorer survival [19]. We have also recently shown that PD-L1 is expressed in the CTCs isolated from NSCLC patients at baseline [20]. Therefore, in this study we focused on the expression of PD-L1 in both groups (treatment naïve and pretreated patients) This investigation, performed in 42 patients from the same group, revealed that similar to VIM expression, PD-L1-positive CTCs were detected more frequently in treatment naïve patients, thus implying possible coordination between EMT status and PD-L1 expression. These results agree with recent studies in which it was demonstrated that PD-L1 is primarily expressed in untreated patients [20,34]. The above theory is also in line with the study of Kim et al., in which a positive correlation between VIM and PD-L1 expression was observed [43], yet further studies are necessary to assess more thoroughly the relation of these molecules. Interestingly the expression of PD-L1 in the examined cohort of patients was related to poorer OS (*p* = 0.035) in agreement with previous studies [44].

Despite these interesting results, the limitation of this work is the pilot nature of the study, including a small number of patients. Further studies will be needed to confirm the clinical relevance of all the examined molecules for patients with NSCLC.

## 5. Conclusions

In conclusion, TUB, GLU, VIM and PD-L1 were highly expressed in NSCLC patients’ CTCs. These results are in line with the previous study in breast cancer patients. The upregulation of these biomarkers could also represent potential prognostic factors for lung cancer patients. Conversely, the absolute number of CTCs, irrespective of the distinct phenotypes, was not correlated to patients’ outcomes, implying that the characterization could be more important than the enumeration of CTCs.

## Data Availability

Data used and analyzed for the current study are available from the corresponding author on reasonable request.

## Supplementary Materials

The following are available online at https://www.mdpi.com/article/10.3390/jpm12020154/s1, Figure S1: Clinical outcome in NSCLC patients. (a) Overall survival (OS) in patients harboring TUB-expressing CTCs; (b) Overall survival (OS) in 1st line patients harboring TUB-expressing CTCs; (c) Overall survival (OS) in advanced patients harboring CTCs with (CK+TUBlowCD45−) phenotype; (d) Overall survival (OS) in advanced patients harboring CTCs with high VIM expression, (e) Overall Survival in patients with PD-L1 positive CTCs in their blood; Table S1: Number of CTCs per phenotype and per patient; Table S2: Patients’ characteristics.
