# Supplementary material for: Detyrosinated α-Tubulin, Vimentin and PD-L1 in Circulating Tumor Cells (CTCs) Isolated from Non-Small Cell Lung Cancer (NSCLC) Patients
Source: J Pers Med. 2022 Jan 25;12(2):154. doi: 10.3390/jpm12020154 (PMC8875112; doi:10.3390/jpm12020154)

Table S1. Patients' Characteristics.

Treatment-naïve (1st line)		Pre-treated (advanced disease)	
Number of patients enrolled	33	Number of patients enrolled	27
Age, years, median (range)	64 (44–81)	Age, years, median (range)	62 (32–80)
	N (%)		*N* (%)
Sex		Sex	
Female	5 (15.2%)	Female	6 (22.2%)
Male	28 (84.8%)	Male	21 (77.8%)
Stage		Stage	
IV (M1a)	5 (15.2%)	IV (M1a)	4 (14.8%)
IV (M1b)	5 (15.2%)	IV (M1b)	11 (40.8%)
IV	4 (12.1%)	IV	4 (14.8%)
IIIA (T3N2M0)	1 (3%)	Unknown	8 (29.6%)
IIIB (T4)	1 (3%)		
Unknown	17 (51.5%)		
Histology		Histology	
Squamous cell carcinoma	6 (18.2%)	Squamous cell carcinoma	7 (26%)
Adenocarcinoma	9 (27.3%)	Adenocarcinoma	13 (48.1%)
Sarcomatoid carcinoma	2 (6%)	Low differentiation squamous	1 (3.7%)
Unknown	16 (48.5%)	Undifferentiated	2 (7,4%)
		Large cell adenocarcinoma + bronchocellular	1 (3.7%)
		Unknown	3 (11.1%)
HLA-A2 status		HLA-A2 status	
Positive	6 (18.2%)	Positive	11 (40.7%)
Negative	9 (27.3%)	Negative	14 (51.9%)
Unknown	18 (54.5%)	Unknown	2 (7.4%)
EGFR status		EGFR status	
WT	9 (27.3%)	WT	16 (59.3%)
Unknown	24 (72.7%)	Unknown	11 (40.7%)
		Line of treatment	
		First	1 (3.7%)
		Second	7 (25.9%)
		Third	9 (33.3%)
		≥ Fourth	8 (29.6%)
		Unknown	2 (7.5%)

Figure S1.

Suppl. Figure 1

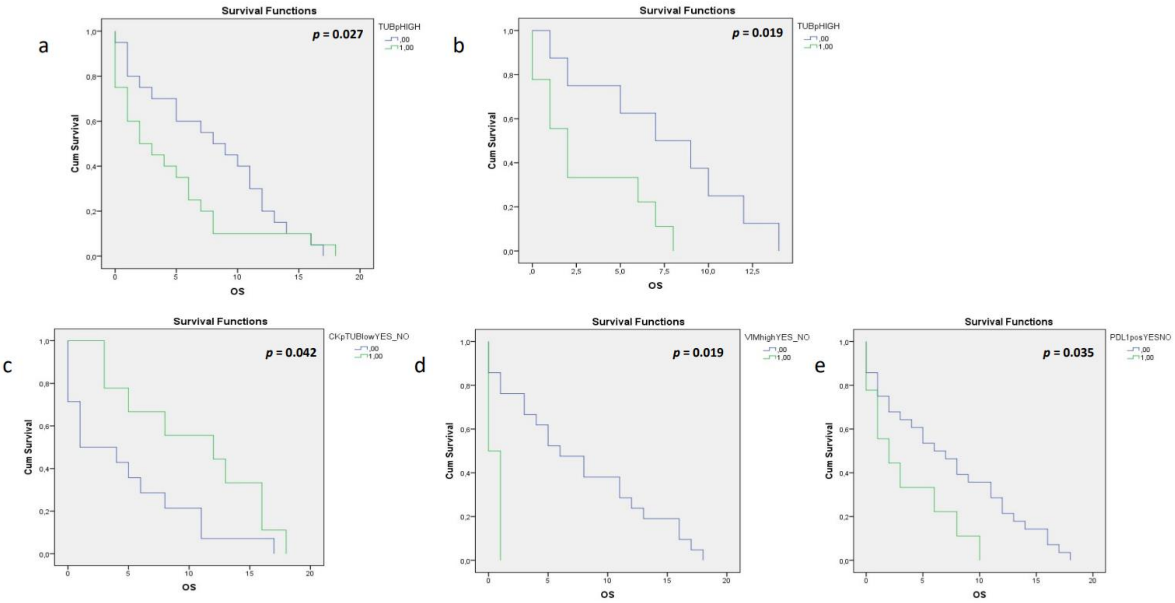

Supplement: Supplementary file 1 [file jpm-12-00154-s001.zip › jpm-1475512-supplementary.pdf]
